# The utility of self-emulsifying oil formulation to improve the poor solubility of the anti HIV drug CSIC

**DOI:** 10.1186/1742-6405-10-14

**Published:** 2013-05-31

**Authors:** Nicholas C Obitte, Lisa C Rohan, Christianah M Adeyeye, Michael A Parniak, Charles O Esimone

**Affiliations:** 1 Department of Pharmaceutical Technology and Industrial Pharmacy, Faculty of Pharmaceutical Sciences, University of Nigeria, Nsukka, Nigeria; 2 Department of Pharmaceutical Sciences, School of Pharmacy, Magee Womens Research Institute, University of Pittsburgh, Pittsburgh, PA, USA; 3 Department of Biopharmaceutical Sciences, College of Pharmacy, Roosevelt University Schaumburg, Shaumburgh, IL, USA; 4 Department of Microbiology and Molecular Genetics, School of Medicine, University of Pittsburgh, Pittsburgh, PA, USA; 5 Department of Pharmaceutical Microbiology and Biotechnology, Faculty of Pharmaceutical Sciences, Nnamdi Azikiwe university, Awka, Anambra, Nigeria

**Keywords:** Anti-HIV, Poorly soluble, Self-emulsification, CSIC, Bioactivity, Anhydrous emulsion

## Abstract

**Background:**

CSIC (5-chloro-3-phenylsulfonylindole-2-carboxamide), a non-nucleoside reverse transcriptase inhibitor (NNRTI) has not been advanced as a therapeutic anti-HIV candidate drug due to its low aqueous solubility and poor bioavailability.

**Objective:**

The objective of this work was to formulate CSIC into self-emulsifying oil formulations for the purpose of improving its aqueous solubility and evaluating *in vitro* antiretroviral activity.

**Methods:**

CSIC self-emulsifying oil formulations (SEFs) were formulated and evaluated for droplet size, zeta potential, polydispersity index (PDI), viscosity, emulsification time, stability and bioactivity.

**Results:**

Results showed significantly improved solubility of CSIC in the SEFs.The concentration of co-surfactant affected the droplet size, zeta potential and polydispersity index. *In vitro* bioactivity studies showed that the CSIC SEFs retained full anti-HIV activity.

**Conclusion:**

The *in vitro* data from this first attempt to formulate CSIC SEFs suggest that improvement on the aqueous solubility of CSIC through this delivery system may accentuate its antiretroviral effectiveness *in vivo* via bioavailability enhancement. The formulation is therefore intended as an oral anti-HIV agent for prophylactic and therapeutic uses.

## Introduction

Approximately 40% of all new drug entities have poor aqueous solubility characteristics [1]. Specific to the HIV field a number of newly developed drug candidates including several non nucleoside reverse transcriptase are extremely hydrophobic. Poorly soluble drugs tend to exhibit low bioavailability, high intra- and inter-subject variability, and a lack of dose proportionality, which pose a significant challenge when administered per orally [2,3]. Drugs that belong to the Class II Biopharmaceutic classification system are frequently caught up in this web of poor solubility [4]. Enhancement of solubility has been achieved by some drug manufacturers through use of special excipients or formulation carriers that enhance drug dissolution and hence, bioavailability. Several approaches including, solid dispersions, inclusion complexes, nanoparticles or lipid vehicles [4-6] are available as potential formulation options for hydrophobic drug candidates. An additional option is self-emulsifying oil formulation.

Self-emulsifying oil formulation (SEF) is a lipid composition with inherent capability of improving drug solubility and bioavailability. SEF is a thermodynamically stable isotropic mixture of oil, surfactant and co-surfactant, which on gentle agitation in aqueous medium self-emulsifies to yield micro or nano-emulsion [7,8]. SEF, in its native form comes as anhydrous emulsion (emulsion pre-concentrate), but upon dispersion in aqueous medium it transforms into oil-in-water (O/W) or water-in-oil (W/O) micro-emulsion or nano-emulsion which constitutes micro-domains of oil droplets stabilized by the interfacial film created by the surfactant and co-surfactant mixture [9,10].

The advantages of SEF over ordinary oil or surfactant dispersions of drugs include the combination of permeability and/or absorption enhancement, thermodynamic stability (long shelf life), ease of preparation (zero interfacial tension and formation spontaneity), optical isotropy, ease of sterilization via filtration, high surface area, very small particle size (that promotes adherence to membranes) [11] and the lipoid characteristic which promotes drug intactness in the droplet prior to absorption. Long shelf life, ease of scale-up and manufacturing, improvement on dissolution and lymphatic transport of poorly soluble or lipophilic drugs rate them better than other novel colloidal drug delivery systems [7]. In the SEF front there are commercially available SEF-based antiretroviral drugs which, demonstrate the contribution of SEFs in bioavailability enhancement. Norvir® (ritonavir), Fortavase® (saquinavir) and Aptivus® (tipranavir) are antiretroviral drugs in the market which have been formulated based on this approach [12,13]. The dose-limiting side effects associated with hydrophobic antiretroviral drugs may be mitigated if formulated in a SEF. This is because erratic drug release common with poorly soluble drugs [2,3] may sometimes lead to the utilization of excessively high drug dose in traditional formulations. SEF ranks well as a reliable carrier that promotes consistent absorption and bioavailability of hydrophobic drugs [2,14].

NNRTIs such as efavirenz, UC781, dapivirine and 5-chloro-3-phenylsulfonylindole-2-carboxamide (CSIC) are potent tight-binding inhibitors of HIV RT, a property that may enhance their utility in both therapeutic and prophylactic use [15]. Efavirenz is poorly soluble, with a bioavailability of 40-45% and belongs to class II Biopharmaceutic classification system (BCS) [16-18]. Similarly CSIC is poorly soluble and has a limited bioavailability which precludes its further development despite superior *in vitro* cell-protective ability [15,19].

CSIC pretreatment of uninfected cells to protect them from subsequent HIV infection in the absence of exogenous drug, resulted to sequestration of CSIC in the cell plasma membrane [15]. This may be suggestive of possible high *in vivo* membrane permeability [20-22]. Nonetheless, the poor aqueous solubility of CSIC may restrict access to absorption through GIT membrane. In this work SEF was explored as a formulation strategy to overcome the limited aqueous solubility and associated bioavailability limitations of CSIC. This is because it offers a nano-sized non-ionic molecular form of the soluble drug which enhances permeability and absorption. Therefore, the objective of this work was to formulate CSIC into SEFs for the purpose of improving its aqueous solubility and evaluating *in vitro* cell-based antiretroviral bioactivity.

## Materials and methods

### Materials

The excipients used included triacetin (Acros Organics of Fisher Scientific, U.S.A), lauroglycol 90® (propylene glycol monolaurate, Gattefosse, France), and Labrasol® (caprylocaproyl macrogol-8-glycerides, Gattefosse, France). CSIC was obtained from Dr. Michael Parniak at the University of Pittsburgh.

### Pseudo ternary phase diagram construction

Labrasol® (surfactant) and lauroglycol 90® (co-surfactant) ratios were varied at five fixed triacetin (oil) ratios (0.5, 1.0, 1.5, 2.0 and 2.5) to generate 30 different oil/surfactant/co-surfactant homogenous isotropic preconcentrate formulations. Oil (triacetin) concentration was evaluated at levels of 10, 20, 30, 40, and 50%. The remainder of the formulations studied consisted of a combination of surfactant and co-surfactant. Labrasol® (surfactant) and lauroglycol 90® (co-surfactant) were evaluated at levels of 10 to 80%. The titration method was adopted for the determination of self-emulsifying region. A 0.1 mL quantity of the SEF was pipetted into a 10 mL beaker. Drop-wise quantities of Milli-Q water (Millipore, USA) filtered through a 0.22 micrometer filter were introduced into the beaker until a stable transparent system was formed. SEF formulations resulting in phase separation or non-transparent emulsions were discarded. The different amounts of the ingredients that contributed to transparent SEFs were determined and a phase diagram plotted using JMP version 4.0.4 software (SAS Institute Inc).

### Assay of CSIC

A liquid chromatography method was used to assay CSIC. A Waters Acquity UPLC was employed. A 1 mg quantity of CSIC was dissolved in 5 mL of Acetonitrile. From the stock solution serial dilutions were made to attain the concentration range of 10, 20, 50, 100 and 150 μg/mL respectively. The corresponding Area under the curve values and the resultant calibration curve were determined using the UPLC machine (Acquity UPLC,Waters USA). CSIC was separated with a C18 (2.1 × 50 mm, 1.7 micrometer) column using a mobile phase of 0.05 v/v% trifluoroacetic acid (TFA) in acetonitrile and 0.08 v/v% of TFA in water at a flow rate of 0.4 ml/min. UV detection was at 302 nm. CSIC eluted at 6 min under these conditions.

### Solubility of CSIC in triacetin

An excess quantity of CSIC was introduced into a vial containing triacetin (oil). The suspension was first vortexed for 10 min and subsequently mixed intermittently for 5 h using a mechanical shaker, prior to storage for 24 h. Using a 0.1 micrometer filter (Fisher Scientific, USA) 20 μL of the suspension was double-filtered, diluted to 1 mL with acetonitrile and assayed for CSIC content using the UPLC method described above. A total of seven replicate samples were analyzed.

### Differential scanning calorimetry

A 2–10 mg quantity of CSIC powder was sealed in a small aluminum pan using a mechanical punch (Perkin Elmer, USA). A sealed empty pan served as control over the temperature range 30 - 400°C. The samples were analyzed using the software, Pyris Series- DSC 7. DSC enabled the determination of melting point of CSIC.

### Formulation of CSIC SEFs

From the experiment described under pseudo ternary phase diagram section the optimal SEF formulations were identified as those which did not result in phase separation and maintained transparency. These formulations are detailed in Table 1. CSIC was combined with each of these formulations at the ratio 0.5 mg CSIC/0.3 ml SEF. Briefly, 0.5 mg CSIC was mixed with triacetin, and then the appropriate amounts of surfactant and co-surfactant were added sequentially with stirring until a homogenous mix was achieved.

**Table 1:** The % concentrations (w/w) of the different components in stable and transparent emulsions

**Formulation**	**Oil:surfactant:cosurfactant**	**Oil (% w/w)**	**Surfactant/cosurfactant mix (%w/w)**	**Water (%w/w)**
A	10:80:10	1.2	8.6	90.2
B	10:70:20	1.8	12.5	85.7
C	20:70:10	2.7	8.5	88.8
D	20:60:20	2.7	8.4	88.9
E	30:60:10	5.4	9.8	84.8

### Characterization of CSIC SEFs

The content of CSIC in each formulation was determined. In these studies a 0.3 mL quantity of SEF was sampled from each of the five formulations described in Table 1 and diluted to 1 mL with acetonitrile. After shaking to ensure complete mixing, the mixtures were assayed for CSIC content using the described UPLC method. Each experiment was carried out in triplicates. Additionally viscosity, droplet size, polydispersity index and zeta potential were also determined for each formulation. Viscosity was measured using 0.5 ml samples with a DV-111 ultra programmable Rheometer (Brookfield Engineering Labs, USA) equipped with a CPE 51 Spindle. Data were calculated with Rheocalc V3.1-2 software. Triplicate determinations were made. A Malvern Zeta Sizer (Malvern Instruments Ltd., UK) was used to determine droplet size, polydispersity index and zeta potential. In these studies transparent emulsions were prepared by gentle agitation of the anhydrous SEF with an appropriate quantity of water. Droplet size was evaluated using cuvette containing 2.5 ml of the nano/microemulsion, and zeta potential was measured using 1 ml in capillary cuvettes. All measurements were performed in triplicates.

### Stability studies for CSIC SEFs

Stability testing for each of the CSIC formulations was evaluated. In these studies each of the CSIC formulations was introduced into vials and stored under three different conditions: (1) 25°C and 60% relative humidity (RH) using a Caron 6010 Humidity Chamber, (2) 40°C and 86% RH using a Caron 6010 Humidity Chamber, and (3) 50°C (Former Scientific Inc., U.S.A). Drug content was determined weekly over a four week period. Additionally stability under stressed conditions was evaluated. Firstly, the effect of centrifugation was studied to evaluate potential metastable conditions, including phase separation and/or drug precipitation. This was done by centrifuging each CSIC formulation at 2000 rpm for 30 min and visually observing for phase separation and drug precipitation. Secondly, the formulations were also stressed by temperature cycling. In brief, the formulations were subjected to a cycle of 12 h refrigeration (4°C) and 12 h storage at room temperature (25°C) for a period of one week. Formulations were then evaluated visually for phase separation or drug precipitation.

### Emulsification time

An adaptation of the method of Koo et al. [23] was used. A 0.3 mL quantity of the SEF was introduced into a beaker containing 200 mL milli-Q water at 37°C. The sample was stirred and visually monitored to determine the time for complete emulsification.

### Drug release/dispersion studies

Drug release/dispersion was measured using a Sotax dissolution apparatus (Sotax CP 7, USA). The dissolution chambers were filled with 100 mL 0.1 N HCl. Analyses were carried out at 37°C and drug content evaluated at 3 min intervals over 40 min.

### Bioactivity testing

Two types of bioactivity tests were carried out, (i) standard antiviral assessments in which cells were simultaneously exposed to varying concentrations of drug and HIV, with drug being present throughout the infection process, and (ii) protective or memory effect assessments in which cells were pretreated with varying concentrations of drug for 16 h, then exogenous drug was removed by extensive washing and the cells exposed to HIV in the absence of exogenous drug. HIV replication was evaluated in single replication cycle HIV assays, using P4R5 HIV infection indicator cells (from Dr. John Mellors, University of Pittsburgh). Cells were maintained in DMEM/10% FBS supplemented with puromycin (0.5 g/mL). P4R5 cells express CD4, CXCR4 and CCR5 as well as a β-galactosidase reporter gene under the control of an HIV LTR promoter [24]. Viral infectivity was assessed in 96-well micro-plate assays using P4R5 cells (5x10^3^cells/well). Cells were inoculated with 25 ng HIV-1 p24/well and the extent of infection was evaluated 48 h post-infection using fluorescence-based β-galactosidase detection assay. Briefly, infected cells were washed, then incubated with 100 L lysis buffer (60 mM Na_2_HPO_4_, 40 mM NaH_2_PO_4_ (pH 7.2), 1 mM MgSO_4_, 100 mM -mercaptoethanol, 2% [v/v] Triton X-100) for 1 h at 37°C. β-Galactosidase activity was assessed by addition of 50 L 4-MUG to a final concentration of 0.5 mM, incubation for 1 h at 37°C, and then quenched with 150 L 0.2 M Na_2_CO_3_, pH 11.2. Fluorescence intensity was assessed with a SPECTRA max GEMINI XS dual-scanning micro-plate spectrofluorometer (Molecular Devices, Sunnyvale, CA) using an excitation wavelength of 355 nm and an emission wavelength of 480 nm, with cutoff filter set to 475 nm.

### Statistics

Results were presented as the mean ± standard deviation. One-way analysis of variance (ANOVA) was employed in the determination of statistical significance using Graph Pad Instat Demo (Graph pad software, Inc., USA). P < 0.05 was considered statistically significant.

## Results

### Pseudo ternary phase diagram

**Figure 1:**
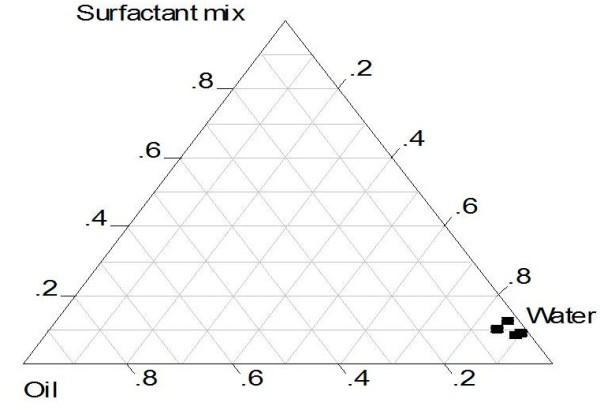
Pseudo-ternary phase diagram for oil, surfactant mix and water.

Of the 30 formulations tested, only five (A-E) (Table 1) formed transparent micro/nano-emulsions without phase separation. These were evaluated as potential SEFs for CSIC. The pseudo ternary phase diagram is an important preliminary strategy adopted in SEFs to delineate self-emulsifying regions [11]. Preparation of SEFs without a preliminary phase diagram construction may result in loss of formulation stability after a period of storage. The pseudo ternary phase diagram is shown in Figure 1.

### Solubility, stability and drug content

The mean solubility of CSIC in triacetin was 0.75 ± 0.04 mg/mL. This limited the unit dose of the formulation to 0.5 mg CSIC. Solubility assessments in the surfactants were not done, since the triacetin oil component was the primary determinant for the drug to remain intact in the droplet post-emulsification. The drug content of the SEFs was between 0.42-0.48 mg (83-93%) (Table 2). Studies to evaluate the effect of temperature and relative humidity on the stability of CSIC in the SEFs showed no substantial changes in CSIC content over the 4 week duration of the experiment (Table 3). Differential scanning calorimetry of CSIC showed a melting peak of 243.67°C.

**Table 2:** Drug content (% CSIC recovery) in various SEFs

**Formulation**	**Oil:surfactant:cosurfactant**	**Drug assay content**	
		**(mg)**	**% recovery**
A	10:80:10	0.468 ± 0.0006	93.62
B	10:70:20	0.436 ± 0.0028	87.12
C	20:70:10	0.453 ± 0.002	90.58
D	20:60:20	0.449 ± 0.0002	89.88
E	30:60:10	0.420 ± 0.001	83.98

**Table 3:** Thermal stability of CSIC in CSIC SEF

		**Amount of CSIC remaining (mg)**								
**FC**^**1**^	**Week 0**	**Week 2**			**Week 3**			**Week 4**		
		**25°C**	**40°C**	**50°C**	**25°C**	**40°C**	**50°C**	**25°C**	**40°C**	**50°C**
A	0.47	0.40 ± 0.01	0.45 ± 0.002	0.48 ± 0.02	0.36 ± 0.001	0.37 ± 0.01	0.39 ± 0.03	0.39 ± 0.04	0.47 ± 0.01	0.49 ± 0.03
B	0.44	0.40 ± 0.008	0.45 ± 0	0.50 ± 0.1	0.35 ± 0.01	0.37 ± 0.02	0.40 ± 0.01	0.39 ± 0.02	0.47 ± 0.02	0.47 ± 0.02
C	0.45	0.414 ± 0.001	0.50 ± 0.01	0.50 ± 0.1	0.36 ± 0	0.39 ± 0.01	0.38 ± 0.02	0.40 ± 0.003	0.48 ± 0.003	0.46 ± 0.02
D	0.45	0.42 ± 0.004	0.45 ± 0.01	0.46 ± 0.01	0.34 ± 0.02	0.39 ± 0.02	0.4 ± 0.01	0.42 ± 0.004	0.46 ± 0.01	0.50 ± 0.001
E	0.42	0.39 ± 0.001	0.44 ± 0.04	0.43 ± 0.01	0.34 ± 0.01	0.36 ± 0.01	0.38 ± 0.01	0.39 ± 0.001	0.44 ± 0.002	0.47 ± 0.005

1 = Formulation code.

### Droplet size, polydispersity index, zeta potential, viscosity and emulsification time

One-Way Analysis of Variance (ANOVA)-based Tukey-Kramer multiple comparisons Test showed that the droplet sizes of placebo and drug-loaded formulations A, C and E respectively were significantly (p < 0.05) smaller than those of formulations B and D (Table 4). Furthermore ANOVA-based Student-Newman-Keuls Multiple Comparisons Test revealed that drug-loading did not significantly (p < 0.05) affect the droplet sizes of the formulations in comparison with placebo batches (Table 4). Placebo formulation D showed significantly (p < 0.05) higher PDI values (Table 4) than those of A, C and E, while B was only significantly (p < 0.05) higher than C and E. Drug-loaded formulations A, C and E recorded significantly (p < 0.05) lower PDI than B and D. On the contrary, drug-loading did not significantly (p < 0.05) contribute to increased PDI. Low polydispersity indices are preferable as emulsions with higher values may be prone to instability. Negative zeta potential often associated with oil-in-water emulsions was generally the case, with minor variations amongst the batches. The viscosity values of the SEFs were 51mpa.s, 48.8mpa.s, 44.3mpa.s, 37.8mpa.s, and 35.7mpa.s for formulations A through E respectively. All formulations displayed a Newtonian flow pattern. The rate of emulsification was too fast (within a few seconds) to be accurately measured. The high rate of emulsification precluded determination of a dissolution profile, as the formulations showed 100% drug release within the earliest time point measured (3 minutes).

**Table 4:** **Droplet size**, **zeta potential and polydispersity index values for the nano**/**micro**-**emulsions**

^**1**^**FC**	**Placebo nano/micro-emulsion**			**Drug-loaded nano/micro-emulsion**		
	**Droplet size (nm)**	**Polydispersity index**	**Zeta potential (-)(mV)**	**Droplet size (nm)**	**Polydispersity index**	**Zeta potential (-)(mV)**
A	40.36 ± 0.69	0.159 ± 0.001	0.635 ± 0.03	67.84 ± 1.6	0.283 ± 0.001	0.635 ± 0.01
B	296.5 ± 54	0.432 ±0.16	0.979 ± 0.002	269 ± 6.1	0.533 ± 0.003	1.58 ± 0.07
C	41.1 ± 0.23	0.134 ± 0.01	1.32 ± 0.02	58.6 ± 0.3	0.283 ± 0.004	1.72 ± 0.04
D	288.5 ± 13	0.559 ± 0.02	1.51 ± 0.01	286 ± 11	0.587 ± 0.001	2.18 ± 0.08
E	52.8 ± 1.4	0.136 ± 0.005	0.970 ± 0.002	65.39 ± 0.9	0.251 ± 0.003	2.25 ± 0.02

1 = Formulation code.

### Bioactivity studies

**Figure 2:**
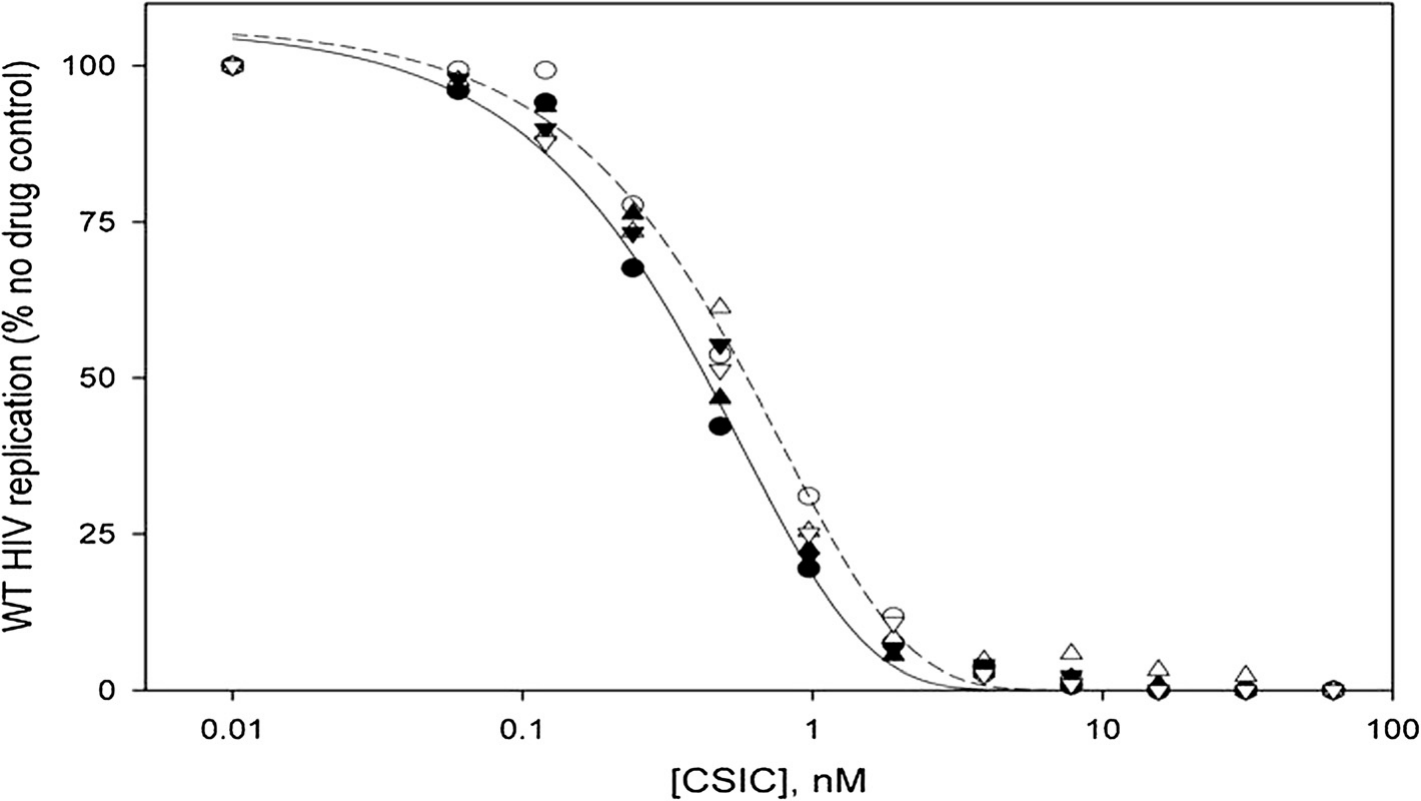
Antiviral activity of CSIC powder and CSIC SEF.

In order to establish that the formulation did not result in loss of bioactivity, *in vitro* anti HIV testing was conducted using the five formulations (A-E). Figure 2 illustrates the antiviral activity for each of the formulations tested. This data showed that the formulations did not result in any loss of antiretroviral bioactivity when compared to unformulated drug substance. Furthermore, the highest concentration tested in these experiments was 0.5 μM. No toxicity was seen at this level indicating that the SEFs did not result in increased toxicity at the 0.5 μM level of dosing. The “memory effect” of the SEFs at 0.1, 1.0, and 10 μM was also tested. Cells were incubated with these concentrations for 16 h, then washed and exposed to HIV. All SEFs were very toxic at 10 μM, and partially toxic at 1 μM. CSIC alone was not toxic at any of these concentrations. All CSIC samples either tested unformulated or in SEFs gave complete protection without discernible toxicity at 0.1 μM. The potent protective or memory effect exerted by the SEFs at 0.1 μM showed that the cells maintained ready access to the formulated drug, essentially similar to that of the free unformulated CSIC. On the contrary when the placebo SEFs, were tested there was no anti-HIV activity.

## Discussion

In this study it was found that higher oil and lower surfactant ratios (eg, formulation D) provided a narrow transition window from anhydrous to transparent nano-emulsion than did lower oil and higher surfactant ratios (eg, formulation A). Figure 1 clearly portrays a narrow self-emulsifying region, since only a few formulations formed stable micro/nano-emulsions upon titration with water. Out of the 30 batches evaluated only 5 formed transparent emulsions upon aqueous dilution. The rest either witnessed phase separation or formed nontransparent emulsions with outrageous droplet sizes. Thus they were excluded. Our choice of the five stable batches was for comparative purposes.

The determination of the amount of drug that could be dissolved in the anhydrous emulsion is crucial to avoid post-formulation drug precipitation either during storage or following *in vivo* dispersion in the aqueous GIT environment [21,25]. CSIC was found to dissolve very slowly, but once dissolved in the anhydrous emulsion it did not precipitate out. Dissolution in SEF involves molecular interaction with the excipients that renders the crystalline drug amorphous [26]. The 0.75 mg solubility in triacetin motivated the choice of 0.5 mg per dose of SEF. Our bioactivity studies results have confirmed 0.5 mg as pharmacologically effective. However, establishment of minimum effective concentration and unit dose is anticipated in our next investigation.

**Figure 3:**
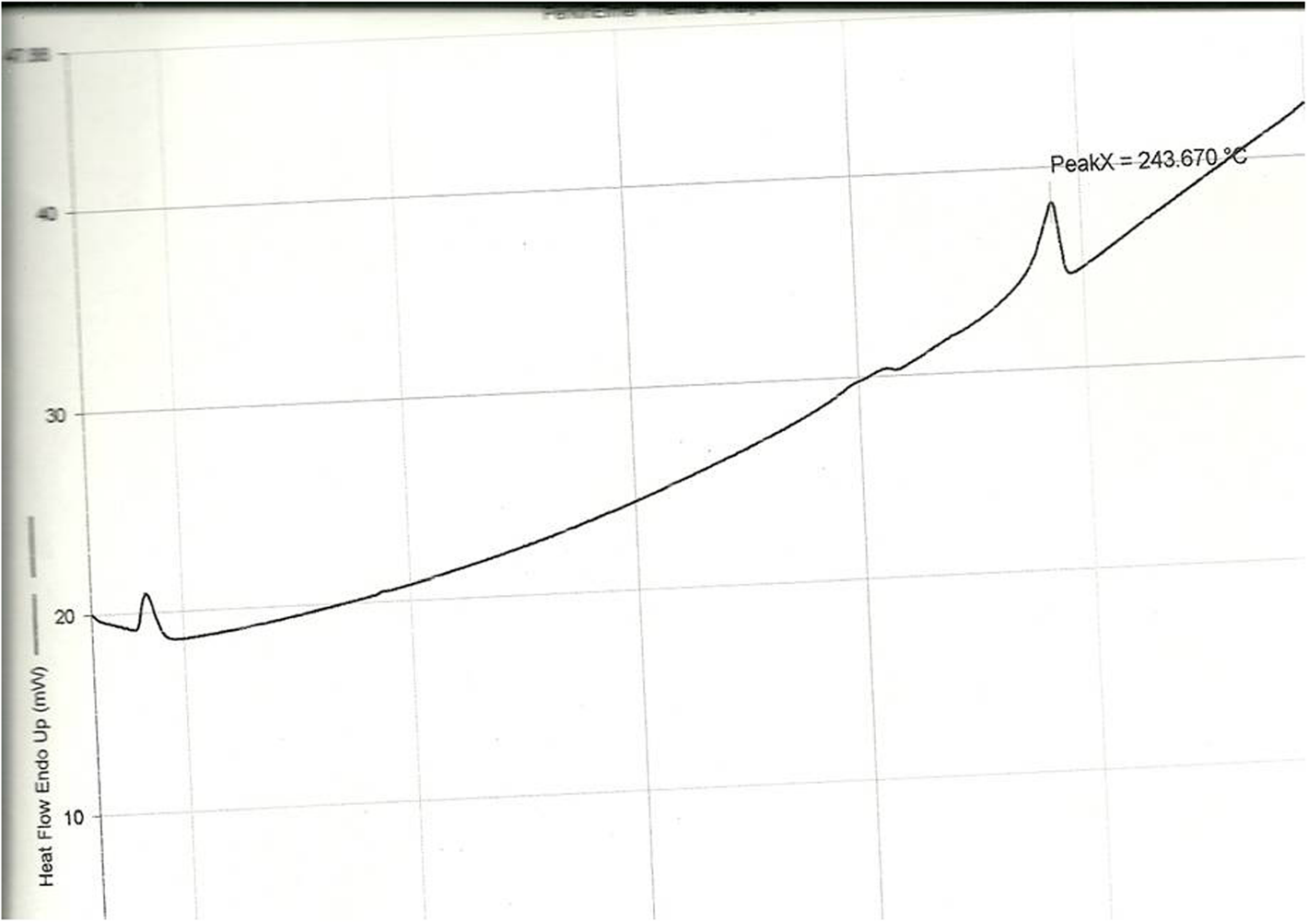
DSC thermogram for CSIC.

Experimental evaluations of the thermodynamic stability of SEFs are important to assess the possibility of drug precipitation or phase separation after centrifugation or repeated refrigeration/warming cycles. With conventional emulsions, the index of stability is the absence of phase separation after centrifugation [27], but in SEFs an additional organoleptic index includes the presence/absence of drug precipitate. All the SEFs maintained thermodynamic stability both in the centrifugation and refrigeration/warming cycle protocols. This may be a clue that this formulation strategy is superior to macro-emulsions or colloidal formulations [5,11]. Furthermore at different temperature and humidity conditions CSIC demonstrated stability in the SEFs. The high melting point of CSIC (Figure 3) was an indication of thermal stability or non-heat sensitivity. This may be why stability studies lacked evidence of thermal drug degradation.

In all the formulations, as the concentrations of oil and surfactant were simultaneously increased and decreased respectively, co-surfactant was held at a fixed concentration of either 10% (formulations A, C, E) or 20% (formulations B, D) (Table 1). It thus appears that droplet size may be related to the co-surfactant concentration used in the formulation; larger droplet sizes resulted from 20% co-surfactant concentration. The contribution of co-surfactant to droplet size may be connected to its supportive functionality during emulsification, where it stabilizes the interfacial role of the surfactant. In micro-emulsions, the interfacial tension is so low that the interfacial energy assumes a similar or lower value than the entropy of dispersion. This renders the free energy zero or negative [28,29]. The use of only one surfactant may hardly achieve transient negative interfacial tension and fluid interfacial film; hence the need for incorporation of a co-surfactant [30]. Generally, co-surfactants lower the interfacial bending stress and consequently predispose the interfacial film to possess enough flexibility to assume various curvatures required for nano/micro-emulsion formation over a wide range of compositions [11,31]. In addition, lauroglycol 90® has a potential dual role of co-surfactant and oil. Higher co-surfactant concentration may have simultaneously increased the interfacial thickness and curvature flexibility of the emulsion droplets with consequent size enlargement. Although the three constituents were miscible, upon emulsification in aqueous phase the cosurfactant with low HLB as the oil may have had better interaction with the oil than the surfactant (with high HLB) and the oil. The polar head of the surfactant may have protruded towards and interacted more with the aqueous medium.

During emulsification more work and energy may be required for droplet formation in high drug-loaded than placebo formulations. Apparently the placebo formulations may ultimately offer lower resistance in aqueous medium during the emulsification process and form smaller droplets than the drug-loaded ones. However in this present investigation the low drug loading (0.5 mg/dose) did not impel significant droplet size or PDI increase [32] probably because little resistance may have been posed.

The oil constituent of the SEFs may be responsible for the negative zeta potential of the nano/micro-emulsions [33]. The viscosity of SEFs is critical during dispersion in aqueous phase. Higher viscosities tend to slow down the emulsification rate which may be detrimental when faster *in vivo* drug release and smooth post-absorption bioavailability profiles are crucial. The low values recorded by the SEF formulations in the present study were low enough to preclude the possibility of erratic or slow self-emulsification.

Self-emulsification occurs when the entropy change favoring dispersion is more than the energy needed to increase the surface area of the dispersion [34,35]. Two minutes has been suggested as an upper acceptable limit for emulsification time [23]; thus the SEFs of the present work were well within this limit. High emulsification rate was responsible for 100% drug release within three minutes.

For an orally administered drug, gastrointestinal and pharmaceutical barriers must be overcome before absorption can take place. Aqueous solubility of the drug in question is a crucial factor because only drug in solution is destined for possible absorption [26]. Since the dissolution rate is the rate-determining step to absorption of poorly soluble drugs, improvement on aqueous solubility will promote absorption and bioavailability [4,36]. Consequently, only the relative quantity of drug that emerges after systemic absorption may be available at the receptor site for pharmacological activity. The bioactivity investigation conducted within this study attempted to predict the absorption status of CSIC. It did not account for the *in vivo* gastrointestinal interactions between drug, food and aqueous GIT fluid. The unformulated CSIC had similar antiviral activity as the CSIC SEF formulations. However the CSIC powder was first dissolved in an aqueous solution of dimethyl sulphoxide (DMSO) since it is poorly water soluble. Otherwise, unaided dispersion in water would be unsolubilised and difficult to acces the cells. This may be the predictable *in vivo* fate when administered orally in that form. The stock solution of the CSIC in DMSO was diluted with excess water and vigorously shaken otherwise drug crystallization would take place. Obviously our SEF formulation offers a less tedious and more commercially viable approach. Two important observations made in our study indicated CSIC SEFs’ therapeutic and prophylactic potentials. Bioactivity established at 0.5 μM and cell protection at, 0.1 μM essentially corroborate drug permeability and activity within the cells. Consequently in the GIT the formulation may witness smooth absorption via the villi of the small intestine. Suffice it to say that of utmost significance was the established improvement on CSIC aqueous solubility which may overcome barrier to its further development as an HIV therapeutic agent [15,19]. Poor water solubility of some antiretroviral drugs may predispose to impaired and inconsistent dissolution within the GIT and consequent erratic absorption. This may cause delay in reaching peak plasma concentration and predispose to sublethal drug concentration. Subpharmacological concentrations may induce resistance. The burden of resistance associated with some hydrophobic antiretrovirals may be due to the above reason.

These studies suggest that this formulation approach could address pharmaceutical product development limitations for the anti-HIV drug candidate CSIC such as poor aqueous solubility and cellular permeability. Furthermore this formulation strategy provides a platform which can be extended to other hydrophobic anti-retroviral agents facilitating their advancement in development.

## Conclusion

Further development of CSIC had been stalled due to poor aqueous solubility and low bioavailability. However, in these studies a self-emulsifying oil formulation has been shown to be a reliable formulation approach for the improvement of CSIC aqueous solubility. The *in vitro* antiretroviral bioactivity of the CSIC SEF formulations developed was also established. We therefore conclude that self-emulsifying oil formulation may provide improved bioavailability for this poorly soluble drug making it a viable drug candidate for evaluation in HIV therapy. Further research works are ongoing in our laboratory. Areas of further investigative concern include use of wider spectrum of surfactants, cosurfactants and oils. Extensive stability and bioavailability studies are also contemplated.

## Competing interests

The authors declare that they have no conflict of interest to declare.

## Authors’ contributions

ONC, RLC, ACM and ECO were involved in the conception, design and preparation of self-emulsifying oil formulations. All the authors were involved in the preparation and editing of the manuscript. PMA designed and carried out the bioactivity studies. All authors read and approved the final manuscript.
